# An Unusual Case of Appendicitis Entrapped Within a Spigelian Hernia

**DOI:** 10.7759/cureus.114778

**Published:** 2026-08-19

**Authors:** Chandler Pugh, Caleb Hudson, Kristina Snoddy, Erin Reid, Stephen Heinzman

**Affiliations:** 1 Medicine, Edward Via College of Osteopathic Medicine, Auburn, USA; 2 Medicine, UAB St. Vincent's East, Birmingham, USA; 3 General Surgery, UAB St. Vincent’s East, Birmingham, USA

**Keywords:** abdominal wall hernia, acute appendicitis, appendix in hernia, atypical appendicitis, postoperative abscess, robotic hernia repair, spigelian hernia

## Abstract

Acute appendicitis within a Spigelian hernia is a rare clinical entity that may present with atypical symptoms, resulting in diagnostic delay and complex operative decision-making. Previously reported cases have most commonly presented with localized right lower quadrant pain, abdominal wall masses, incarceration, bowel obstruction, or strangulation. Spigelian hernias occur along the semilunar line and may contain intra-abdominal structures, including the appendix, resulting in non-classic symptoms of appendicitis.

We report a case of a 52-year-old woman with a history of cerebrovascular accident with residual left-sided weakness, prior laparotomy and colostomy following a gunshot wound to the left lower quadrant (2012), and atrial fibrillation with poor adherence to apixaban, who presented with progressive bilateral lower extremity pain and right lower quadrant pain radiating to the groin, with the atypical lower extremity symptoms initially prompting neurologic and musculoskeletal evaluation and delaying recognition of the underlying abdominal pathology. Initial vital signs were notable for mild tachycardia, and laboratory evaluation demonstrated mild leukocytosis. Over approximately one month, her progressive lower extremity pain was initially attributed to neurologic or musculoskeletal pathology, resulting in nondiagnostic spinal imaging before CT of the abdomen and pelvis established the diagnosis. CT imaging of the abdomen and pelvis ultimately revealed a right Spigelian hernia containing an inflamed appendix, consistent with acute appendicitis within the hernia sac. She underwent robotic appendectomy and hernia repair with mesh placement. Intraoperatively, a suppurative appendix within a 4-cm Spigelian hernia was confirmed. Her postoperative course was complicated by a superficial fluid collection managed with intravenous clindamycin and piperacillin-tazobactam for three days, with subsequent clinical improvement.

This case is notable for the atypical presentation and resulting diagnostic delay. It highlights the importance of considering abdominal wall hernias and anatomic displacement of intra-abdominal structures in patients with persistent groin or lower quadrant pain, particularly when neurologic or musculoskeletal evaluation is unrevealing. Cross-sectional imaging remains essential for identifying uncommon hernia-related causes of appendicitis.

## Introduction

Spigelian hernias are rare ventral hernias that occur through a defect in the anterior abdominal wall adjacent to the semilunar line [1-3]. They most commonly develop in the lower abdominal segment where the posterior rectus sheath is absent [1]. These hernias account for approximately 0.12% of all abdominal wall hernias [1]. Diagnosis is often challenging due to significant variability in clinical presentation. As the hernia sac typically lies beneath the external oblique aponeurosis, a visible or palpable abdominal bulge is often absent. Consequently, patients usually present with nonspecific abdominal, groin, or lower extremity pain. This may obscure the underlying pathology and delay diagnosis [2].

In rare instances, Spigelian hernias may contain the appendix. Published cases of appendicitis within a Spigelian hernia, an exceedingly rare entity with fewer than 20 reported cases, have most commonly presented with localized right lower quadrant pain, abdominal wall masses, incarceration, bowel obstruction, or strangulation and have generally been diagnosed with cross-sectional imaging followed by operative repair [4-8]. Building on the diagnostic complexity outlined above, this report presents a case of a right-sided Spigelian hernia containing an inflamed appendix, with unique comorbidities and an atypical pain distribution. Taken together, these factors contributed to a delay in the initial diagnosis, underscoring the challenges in recognizing this rare entity.

## Case presentation

A 52-year-old female with a history of a cerebral vascular event with residual left-sided weakness, atrial fibrillation, and prior abdominal trauma presented with bilateral lower extremity swelling and pain with declining mobility over approximately one month. During this period, she also developed right lower quadrant abdominal pain radiating to the groin. Her right leg pain progressively worsened her mobility, leaving her wheelchair-bound despite prior ambulation.

The patient denied fever, nausea, vomiting, changes in bowel habits, or urinary symptoms. Her medication history was notable for chronic opioid therapy (oxycodone/acetaminophen 10 mg/325 mg) through a pain management group, which she reported was no longer effective. She had a history of atrial fibrillation but had discontinued apixaban and was taking aspirin alone. Her surgical history was significant for a gunshot wound to the left lower quadrant in 2012, requiring exploratory laparotomy and colostomy formation with later reversal. Vital signs on presentation were: temperature 37.3°C, heart rate 105 beats per minute, respiratory rate 17 breaths/minute, blood pressure 138/94 mmHg, and oxygen saturation 96% on room air.

Physical exam showed bilateral leg edema and tenderness with baseline neurologic deficits. Abdominal exam demonstrated right lower quadrant tenderness without peritoneal signs or masses.

**Figure 1:**
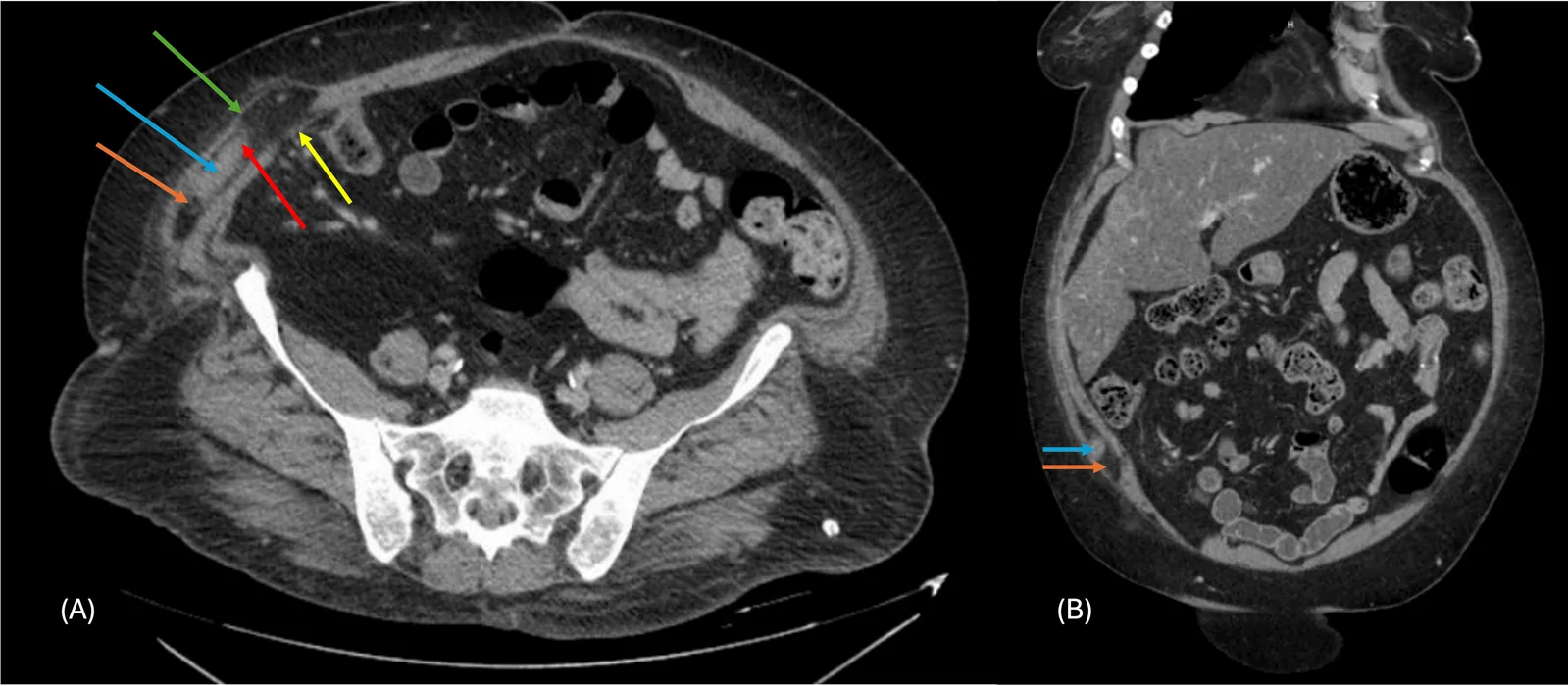
Preoperative CT of the Abdomen and Pelvis with Contrast CT images demonstrating acute appendicitis within a right lower lateral abdominal wall Spigelian hernia. Axial image (A) demonstrates an inflamed appendix (blue arrow) contained within the hernial sac (green arrow). The fascial defect (yellow arrow) and hernia neck (red arrow) are identified at the site of protrusion through the abdominal wall. Surrounding periappendiceal inflammatory fat stranding (orange arrow) is present, consistent with acute appendicitis. Sagittal image (B) further demonstrates the inflamed appendix (blue arrow) with adjacent inflammatory fat stranding (orange arrow) extending within the hernia sac.

Laboratory evaluation demonstrated leukocytosis without anemia or thrombocytopenia and anion-gap metabolic acidosis with an anion gap of 14 (Table 1). Given her prior cerebrovascular accident with baseline neurologic deficits and the predominance of lower extremity symptoms, the initial diagnostic consideration was neurologic or musculoskeletal pathology rather than an intra-abdominal process. Upon presentation, evaluation included computed tomography (CT) imaging of the cervical, thoracic, and lumbar spine, as well as lower extremity venous duplex ultrasonography, all of which were negative for an acute abnormality. Persistent right lower quadrant and groin pain prompted CT imaging of the abdomen and pelvis with and without contrast during the same admission. CT abdomen and pelvis with contrast (Figure 1) was significant for a right lower lateral abdominal wall. Spigelian hernia containing an enlarged, inflamed appendix with surrounding periappendiceal fat stranding. These imaging findings allowed the establishment of acute appendicitis within a Spigelian hernia.

**Table 1:** Laboratory Values on Admission Laboratory values on admission demonstrated mild leukocytosis with anion-gap metabolic acidosis.

Component	Patient Value	Reference Range	Interpretation
Complete Blood Count			
White Blood Cell Count	12.24 × 10³/µL (H)	4.0–11.0 × 10³/µL	Mild leukocytosis
Red Blood Cell Count	4.6 × 10⁶/µL	4.2 – 5.4 × 10⁶/µL	Normal
Hemoglobin	13.7 g/dL	12-16 g/dL	Normal
Hematocrit	40.4%	36-46%	Normal
Mean Corpuscular Volume	87.8 fL	80-100 fL	Normal
Mean Corpuscular Hemoglobin	29.8 pg	25.4-34.6 pg	Normal
Mean Corpuscular Hemoglobin Concentration	33.9%	31-36%	Normal
Red Cell Distribution Width	14.6% (H)	11-15%	Normal
Platelets	237 × 10⁹/L	150–400 × 10⁹/L	Normal
Mean Platelet Volume	9.7 fL	7.5-12.0 fL	Normal
Neutrophils	65.7%	40–70%	Normal
Lymphocytes	21.5%	20-40%	Normal
Monocytes	10.2% (H)	2-8%	Mild monocytosis
Eosinophils	1.4%	1-4%	Normal
Basophils	0.4%	0-1%	Normal
Immature Granulocytes (%)	0.8% (H)	0-0.5%	Elevated
Absolute Neutrophil Count	8.04 x 10^9^/L (H)	1.5-8 x 10^9^/L	Mild neutrophilia
Absolute Lymphocyte Count	2.63 x 10^9^/L	1.0-4.8 x 10^9^/L	Normal
Absolute Monocyte Count	1.25 x 10^3^/uL (H)	0.2-0.95 x 10^3^/uL	Monocytosis
Absolute Eosinophil Count	0.17/uL	0-500/uL	Normal
Absolute Basophil Count	0.05 x 10^9^/L	0-0.2 x 10^9^/L	Normal
Absolute Immature Granulocyte Count	0.10 x 10^9^/L (H)	0-0.03 x 10^9^/L	Elevated
Routine Chemistry			
Sodium Level	142 mEq/L	136-145 mEq/L	Normal
Potassium Level	3.9 mEq/L	3.5-5.0 mEq/L	Normal
Chloride	107 mEq/L (H)	98-106 mEq/L	Mild hyperchloremia
Bicarbonate	21 mEq/L (L)	22-28 mEq/L	Low; Metabolic acidosis
Anion Gap	14.0 mEq/L (H)	6-12 mEq/L	Mildly elevated; High anion gap metabolic acidosis
Blood Urea Nitrogen to Creatinine Ratio	14:1	10-20:1	Normal
Blood Urea Nitrogen	11 mg/dL	10-20 mg/dL	Normal
Glucose Level	106 mg/dL	74-106 mg/dL	Normal
Creatinine Level	0.80 mg/dL	0.8-1.3 mg/dL	Normal
Estimated Glomerular Filtration Rate	89	90+	Slightly reduced
Calcium Level	9.9 mg/dL	9-10.5 mg/dL	Normal
Albumin Level	3.8 g/dL	3.5-5.5 g/dL	Normal
Albumin-to-Globulin Ratio	0.8 (L)	1.1-2.5	Low
Total Protein	8.3 g/dL	6.4-8.3 g/dL	Normal
Alanine Aminotransferase	34 U/L	4-36 U/L	Normal
Aspartate Aminotransferase	37 U/L (H)	0-35 U/L	Mild elevation
Alkaline Phosphatase	118 U/L	3-120 U/L	Normal
Total Bilirubin	< 0.30 mg/dL	0.3-1.0 mg/dL	Low
Other			
Magnesium Level	2.5 mEq/L (H)	1.3-2.1 mEq/L	Mild hypermagnesemia
Total Creatinine Kinase	93 U/L	20-200 U/L	Normal
B-type Natriuretic Peptide	29 pg/mL	<100 pg/mL	Normal

The patient was taken to the operating room for robotic-assisted laparoscopic appendectomy and ventral hernia repair. Following induction of general anesthesia, standard perioperative prophylaxis, including IV cefazolin and venous thromboembolism prophylaxis, was administered. Abdominal access was achieved via a left upper quadrant Veress needle. Right upper quadrant and lateral trocars were then placed.

Intraoperative findings confirmed a 4 cm right-sided Spigelian hernia containing an inflamed appendix. The appendix was suppurative but non-perforated, without fecal contamination, abscess, or diffuse peritonitis, consistent with a contaminated (CDC Class III) operative field. The appendix was carefully dissected free from the hernia sac, the mesoappendix was controlled, and the appendiceal base was divided using an endoscopic stapler. Contamination mitigation strategies included specimen extraction with an endobag and copious irrigation prior to hernia repair. Immediate repair was pursued, given the symptomatic fascial defect and concern for recurrent incarceration. Attempted preperitoneal dissection was unsuccessful; therefore, the fascial defect was primarily closed using 2-0 barbed suture (V-LokTM, Medtronic, Dublin, Ireland) in two layers. In the setting of potential contamination and unsuccessful preperitoneal dissection, 10 × 15 cm biosynthetic hybrid mesh (SynecorTM, W.L. Gore & Associates, Flagstaff, AZ, USA) was selected to reinforce the repair and secured in place. Recovery was uneventful and included two days of inpatient monitoring with administration of intravenous piperacillin-tazobactam 3.375 g every six hours. Following inpatient observation, the patient was discharged in stable condition with a seven-day course of amoxicillin-clavulanate 875/125 mg twice daily.

**Figure 2:**
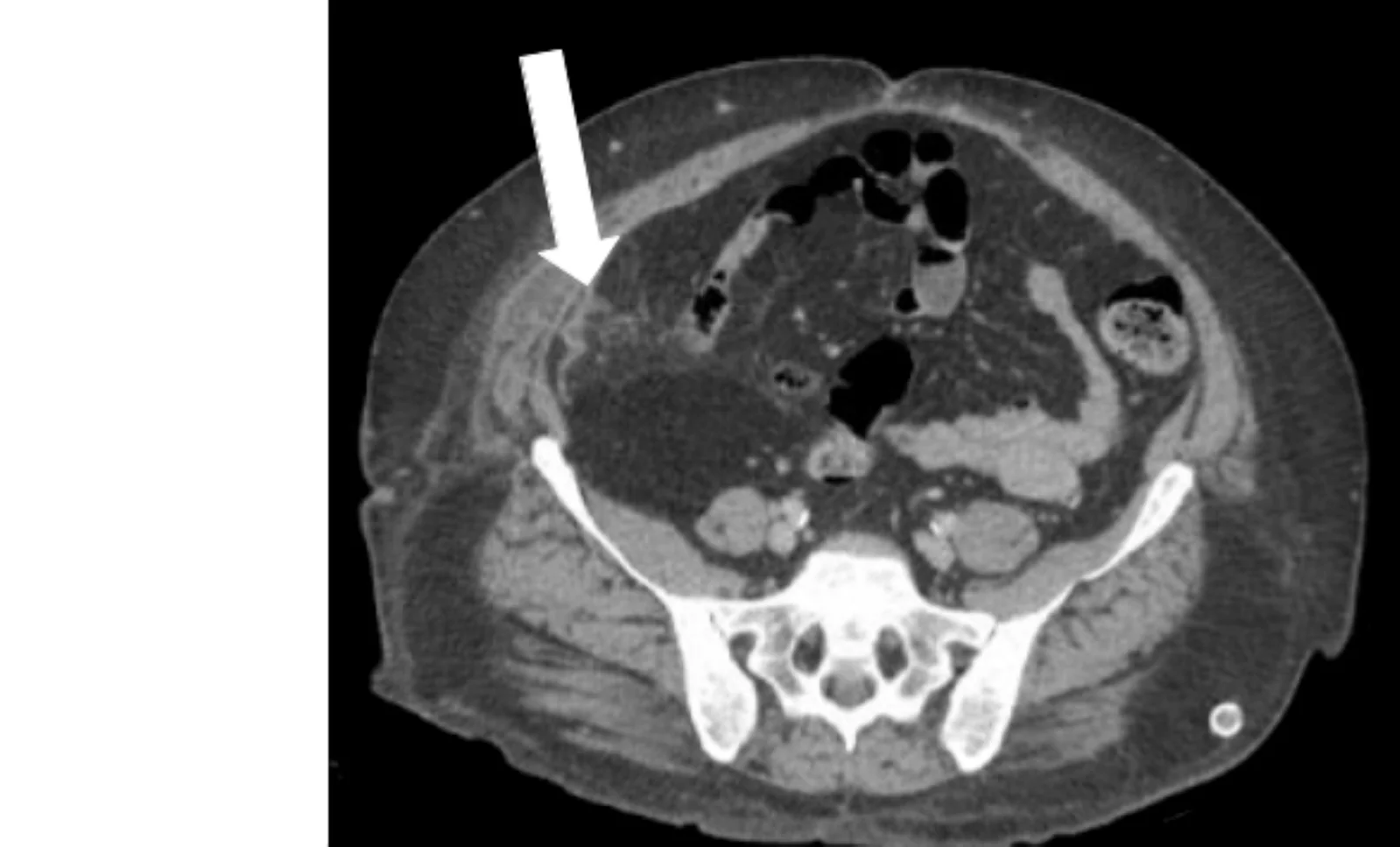
Postoperative CT of the Abdomen and Pelvis with Contrast Axial image obtained two weeks following appendectomy and Spigelian hernia repair with mesh, demonstrating a 3.7 cm superficial wound fluid collection (arrow) with surrounding inflammatory fat stranding. No internal gas, mature rim-enhancing capsule, deeper intra-abdominal extension, or radiographic evidence of communication with the underlying mesh is identified. These findings were concerning for an evolving early infectious collection rather than a mature drainable abscess.

Two weeks postoperatively, the patient returned to the emergency department with acute-onset abdominal pain rated 5/10. Laboratory evaluation was largely unrevealing, but demonstrated monocytosis and anemia (Table 2). She was again evaluated with CT of the abdomen and pelvis with contrast (Figure 2), which demonstrated expected postoperative changes from appendectomy and hernia repair with mesh placement, as well as a 3.7 cm superficial fluid collection overlying the abdominal musculature with surrounding inflammatory stranding, concerning for an evolving early infectious collection rather than a mature drainable abscess. No internal gas, organized rim enhancement, or definite mesh communication was identified. At this point, interventional radiology was consulted and deemed the fluid collection to be too thin to drain. Thus, drainage was deemed not clinically appropriate in this patient. She was admitted and treated with intravenous clindamycin (600 mg/50 mL) and piperacillin-tazobactam (4.5 g/20 mL) for three days, resulting in complete clinical resolution without need for drainage or reoperation. Following this treatment, the patient was discharged home with a prescription of oral clindamycin 300 mg for five days.

**Table 2:** Laboratory Values on Subsequent Admission Two Weeks Later Laboratory evaluation upon return to the emergency department two weeks postoperatively was largely unrevealing, but demonstrated monocytosis and anemia.

Component	Patient Value	Reference Range	Interpretation
Complete Blood Count			
White Blood Cell Count	10.98 × 10³/µL	4.0–11.0 × 10³/µL	Normal
Red Blood Cell Count	4.09 × 10⁶/µL (L)	4.2 – 5.4 × 10⁶/µL	Low
Hemoglobin	12.5 g/dL	12-16 g/dL	Normal
Hematocrit	36.3%	36–46%	Normal
Mean Corpuscular Volume	88.8 fL	80-100 fL	Normal
Mean Corpuscular Hemoglobin	30.6 pg	25.4-34.6 pg	Normal
Mean Corpuscular Hemoglobin Concentration	34.4%	31-36%	Normal
Red Cell Distribution Width	14.4%	11-15%	Normal
Platelets	213 × 10⁹/L	150–400 × 10⁹/L	Normal
Mean Platelet Volume	10.4 fL	7.5-12.0 fL	Normal
Neutrophils	46.8%	40–70%	Normal
Lymphocytes	36.9%	20-40%	Normal
Monocytes	12.1% (H)	2-8%	Monocytosis
Eosinophils	3.1%	1-4%	Normal
Basophils	0.5%	0-1%	Normal
Immature Granulocytes (%)	0.6% (H)	0-0.5%	Mild elevation
Absolute Neutrophil Count	5.13 x 10^9^/L	1.5-8 x 10^9^/L	Normal
Absolute Lymphocyte Count	4.05 x 10^9^/L	1.0-4.8 x 10^9^/L	Normal
Absolute Monocyte Count	1.33 x 10^3^/uL (H)	0.2-0.95 x 10^3^/uL	Monocytosis
Absolute Eosinophil Count	0.34/uL	0-500/uL	Normal
Absolute Basophil Count	0.06 x 10^9^/L	0-0.2 x 10^9^/L	Normal
Absolute Immature Granulocyte Count	0.07 x 10^9^/L (H)	0-0.03 x 10^9^/L	Elevated
Routine Chemistry			
Sodium Level	140 mEq/L	136-145 mEq/L	Normal
Potassium Level	3.7 mEq/L	3.5-5.0 mEq/L	Normal
Chloride	104 mEq/L	98-106 mEq/L	Normal
Bicarbonate	24 mm Hg	22-28 mEq/L	Normal
Anion Gap	12.0 mEq/L	6-12 mEq/L	Normal
Blood Urea Nitrogen to Creatinine Ratio	16	10-20:1	Normal
Blood Urea Nitrogen	13 mg/dL	10-20 mg/dL	Normal
Glucose Level	82 mg/dL	74-106 mg/dL	Normal
Creatinine Level	0.80 mg/dL	0.8-1.3 mg/dL	Normal
Estimated Glomerular Filtration Rate	89	90+	Stable
Calcium Level	9.6 mg/dL	9-10.5 mg/dL	Normal
Albumin Level	3.4 g/dL (L)	3.5-5.5 g/dL	Mild hypoalbuminemia
Albumin-to-Globulin Ratio	0.8 (L)	1.1-2.5	Low
Total Protein	7.9 g/dL	6.4-8.3 g/dL	Normal
Alanine Aminotransferase	70 U/L (H)	4-36 U/L	Elevated
Aspartate Aminotransferase	60 U/L (H)	0-35 U/L	Elevated
Alkaline Phosphatase	137 U/L (H)	3-120 U/L	Elevated
Total Bilirubin	< 0.30 mg/dL	0.3-1.0 mg/dL	Low
Other			
Lipase Level	38 units/L	0-160 units/L	Normal
Amylase	28 units/L	30-220 units/L	Low
Urinalysis			
Urine Color	Yellow	Yellow	Normal
Urine Appearance	Clear	Clear	Normal
Urine pH	5.5	4.6-8	Normal
Urine Leukocyte Esterase	Negative	Negative	Normal
Urine Nitrite	Negative	Negative	Normal
Urine Protein	Negative	0-8 mg/dL	Normal
Urine Glucose	Negative	Negative	Normal
Urine Ketones	Trace (H)	Negative	Mild ketosis
Urine Urobilinogen	0.2 mg/dL	0.2-1 mg/dL	Normal
Urine Bilirubin	Negative	Negative	Normal
Urine Blood	Negative	Negative	Normal
Urine Specific Gravity	1.020	1.005-1.03	Normal

## Discussion

Spigelian hernias are rare ventral hernias, accounting for 0.12% of all abdominal wall hernias [1-3]. These hernias occur through a defect in the Spigelian aponeurosis, located between the lateral edge of the rectus muscle and the semilunar line [3]. The presence of an inflamed appendix within a Spigelian hernia is exceedingly rare, with fewer than 20 cases reported in the literature [4,5]. If the appendix were incarcerated within an inguinal hernia, it would be termed an Amyand hernia; however, in our case, the appendix herniated through the anterior abdominal wall via a Spigelian defect, representing a distinct and rarer clinical entity [4,5].

Previously reported cases of appendicitis within a Spigelian hernia have generally presented with localized right lower quadrant pain, palpable abdominal wall masses, incarceration, bowel obstruction, or strangulation [4-8]. CT imaging has consistently played a central role in diagnosis, while operative management has most commonly involved open or laparoscopic appendectomy with either primary repair or selective mesh reinforcement, depending on the degree of contamination. In contrast, our patient presented with an atypical symptom complex characterized by bilateral lower extremity pain, groin radiation, and progressive functional decline, prompting extensive spinal evaluation and delayed recognition of the underlying abdominal pathology. Prior laparotomy and colostomy reversal increased diagnostic ambiguity and operative complexity. This case also demonstrates the feasibility of robotic-assisted repair with biosynthetic mesh reinforcement in the setting of suppurative but non-perforated appendicitis within a Spigelian hernia. The subsequent postoperative superficial infectious fluid collection, which resolved with intravenous antibiotics alone, further illustrates the challenges associated with mesh use in potentially contaminated operative fields. Collectively, this case expands the limited literature by highlighting an atypical pain distribution, delayed diagnosis due to competing neurologic and musculoskeletal considerations, and complex perioperative decision-making in abdominal wall hernia repair.

Comparison to similar cases

Review of the literature reveals several comparable presentations. Thomas et al. reported appendicitis within a Spigelian hernia presenting emergently as a tender right iliac fossa mass, emphasizing the diagnostic challenge these cases pose [6]. Lin et al. described a patient with right lower quadrant pain due to concurrent incarcerated Spigelian hernia and acute appendicitis, noting that right-sided Spigelian hernias can clinically mimic appendicitis and complicate preoperative diagnosis [7]. Onal et al. reported strangulation of both the small bowel and appendix within a Spigelian hernia, successfully managed with primary repair reinforced by polypropylene mesh [8]. Stanley et al. described laparoscopic repair of a Spigelian hernia with in situ appendicitis, demonstrating the feasibility of minimally invasive management [4]. Our case adds to this limited literature by demonstrating successful robotic-assisted repair with biosynthetic mesh reinforcement in a patient with an atypical pain distribution characterized by bilateral lower extremity pain and right lower quadrant pain radiating to the groin.

Diagnostic considerations

Spigelian hernias are frequently misdiagnosed because of nonspecific symptoms and the absence of an obvious abdominal wall bulge, as they typically occur beneath the intact external oblique aponeurosis [1,2]. Clinical presentation often includes localized pain, a palpable mass, and signs of bowel obstruction when incarceration occurs [1-3]. In this case, the diagnostic challenge was compounded by atypical symptoms, including lower extremity pain and functional decline. Prior abdominal surgery may have further obscured clinical findings and increased susceptibility to hernia formation. These factors underscore the importance of maintaining a broad differential diagnosis for groin, lower quadrant, or lower extremity pain, particularly in patients with complex medical histories or prior abdominal interventions.

The patient’s bilateral lower extremity pain represented a significant diagnostic confounder. Given her prior cerebrovascular accident with residual neurologic deficits and progressive mobility impairment, the pain was initially suspected to reflect neurologic, musculoskeletal, or vascular pathology, prompting multiple nondiagnostic spine imaging studies and vascular evaluation. The absence of classic gastrointestinal symptoms further reduced early suspicion for appendicitis or abdominal wall hernia. Although a direct pathophysiologic relationship between the Spigelian hernia and lower extremity pain cannot be definitively established, it is possible that regional inflammation or nerve irritation from the incarcerated, inflamed appendix contributed to referred groin or leg discomfort. However, this association remains speculative, and the lower extremity symptoms may also have represented unrelated chronic baseline pathology. Regardless, the atypical pain distribution substantially delayed recognition of the underlying abdominal process and highlights the importance of maintaining a broad differential diagnosis in patients with persistent groin or lower quadrant pain despite unrevealing neurologic or musculoskeletal evaluation.

CT imaging proved pivotal in this case, enabling accurate diagnosis and timely surgical intervention. Cross-sectional imaging is considered the gold standard for diagnosing Spigelian hernias, with CT demonstrating high sensitivity for identifying the fascial defect, hernia contents, and any associated complications such as strangulation or obstruction [9]. Early imaging improves recognition of these rare hernias and reduces the risk of delayed diagnosis.

Surgical management

Surgical management of Spigelian hernias can be performed using open, laparoscopic, or robotic approaches. A recent systematic review of 1,629 patients found that minimally invasive approaches were used in 46.8% of cases and were associated with shorter hospital stays, and that open surgery was more commonly utilized in emergency settings [10]. Robotic-assisted repair was used in this case to facilitate management of a rare Spigelian hernia containing an inflamed appendix. Enhanced visualization and wristed instrumentation allowed careful reduction of the appendix from the hernia sac and precise dissection within the confined lateral abdominal wall defect. Robotic assistance also facilitated intracorporeal fascial closure and controlled placement and fixation of biosynthetic mesh despite limited operative space. While these technical advantages were observed in this case, no conclusions can be drawn regarding superiority over laparoscopic or open repair.

Mesh reinforcement is recommended in Spigelian hernia repair to reduce recurrence [11,12]. In this case, unsuccessful preperitoneal dissection necessitated onlay mesh placement. Although contemporary evidence generally favors preperitoneal positioning to reduce adhesions and mesh-related complications, recurrence rates remain low regardless of technique [12]. The totally extraperitoneal approach may further reduce these risks by avoiding peritoneal entry; however, Cui et al. reported comparable outcomes among intraperitoneal onlay mesh, transabdominal preperitoneal, and totally extraperitoneal approaches [13]. Failure to achieve preperitoneal placement reflected the technical challenges of emergency repair with concurrent intra-abdominal pathology, and biosynthetic mesh was selected given the potentially contaminated operative field.

Clinical implications

This case reinforces several important clinical principles. First, atypical pain patterns, especially with concomitant leukocytosis, should prompt consideration of rare hernia presentations such as Spigelian hernias. Second, early cross-sectional imaging is critical to avoid diagnostic delay and guide surgical planning [9]. Third, minimally invasive approaches, including laparoscopic and robotic-assisted repair, can be successfully employed even in complex cases with concurrent appendicitis [4]. Finally, mesh reinforcement remains the standard of care for durable repair, though mesh selection and placement must be individualized to the clinical context [11,12].

The patient presented two weeks postoperatively with abdominal pain and a 3.7 cm superficial fluid collection overlying the abdominal musculature with surrounding inflammatory fat stranding, concerning for early abscess formation. CT imaging demonstrated no internal gas, mature rim-enhancing capsule, deeper intra-abdominal extension, or radiographic evidence of mesh involvement. Given the collection’s small size and superficial location, along with the absence of imaging features suggestive of a mature drainable abscess, management with intravenous clindamycin 600 mg in 50 mL and piperacillin/tazobactam 4.5 g in 20 mL was selected. During the three-day admission, the patient remained hemodynamically stable without systemic sepsis, diffuse peritonitis, or significant leukocytosis and demonstrated rapid clinical improvement after three days of intravenous antibiotic therapy, eliminating the need for drainage or reoperation. Repeat imaging was not obtained because the patient's symptoms resolved clinically, and the patient declined follow-up. This case underscores the importance of close postoperative follow-up after repair of Spigelian hernias containing appendicitis and highlights the management considerations that arise when postoperative fluid collections develop in the setting of mesh reinforcement.

## Conclusions

This case illustrates the diagnostic and therapeutic challenges of appendicitis within a Spigelian hernia, an exceedingly rare entity. The patient's atypical presentation with bilateral lower extremity pain rather than classic abdominal wall findings underscores the importance of maintaining a broad differential diagnosis, particularly in patients with prior abdominal surgery and complex comorbidities. CT imaging proved essential for accurate preoperative diagnosis, enabling timely surgical intervention before bowel compromise.

Robotic-assisted repair with mesh reinforcement was successfully performed despite technical challenges, including suboptimal onlay mesh placement due to unsuccessful preperitoneal dissection. The postoperative abscess was successfully managed with intravenous antibiotics without requiring drainage or reoperation, demonstrating that early recognition and conservative treatment can effectively address infectious complications. This case reinforces that Spigelian hernias require high clinical suspicion and early cross-sectional imaging to avoid diagnostic delays, and that minimally invasive approaches can achieve favorable outcomes even in complex presentations with concurrent appendicitis.
